# Pulmonary Manifestation of Granulomatosis With Polyangiitis: A Challenging Case Presentation

**DOI:** 10.7759/cureus.57515

**Published:** 2024-04-03

**Authors:** Chaynez Rachid, Mohamed Ijim, Oussama Fikri, Lamyae Amro

**Affiliations:** 1 Pulmonology Department, Laboratoire de Recherche Morpho Sciences, Faculté de Médecine et de Pharmacie de Marrakech, Université Cadi Ayyad, Centre Hospitalier Universitaire (CHU) Mohammed VI, Hôpital Arrazi, Marrakech, MAR

**Keywords:** corticosteroides, thoracic radiology, diffuse alveolar hemorrhage, diffuse interstitial lung disease, ground-glass opacity

## Abstract

Granulomatosis with polyangiitis (GPA), formerly known as Wegener's granulomatosis, is a rare and potentially life-threatening autoimmune disease characterized by antineutrophil cytoplasmic antibody (ANCA)-associated vasculature inflammation. It presents as a systemic autoimmune disease with necrotizing granulomatous inflammation and pauci-immune small vessel vasculitis. This case initially posed a diagnostic challenge due to its atypical presentation and was misdiagnosed as hypersensitivity pneumonitis. The avian precipitin screening assay was positive in our patient, which may be consistent with bird breeder's lung disease or a non-specific reactivity of the chicken antigen test. However, the presence of positive c-ANCA was pivotal for the GPA diagnosis. Here, we describe in detail the clinical manifestations, diagnostic approach, and treatment of GPA in a 54-year-old female who presented with alveolar hemorrhage, but no renal involvement. Treatment involved the use of high-dose corticosteroids to suppress the autoimmune response. Finally, we discuss the striking response of this unique form of granulomatosis with polyangiitis to corticosteroid treatment and emphasize the importance of early initiation of treatment.

## Introduction

Granulomatosis with polyangiitis (GPA), formerly known as Wegener's granulomatosis, is an unknown systemic inflammatory disease that affects the kidneys, upper and lower respiratory tracts, and small vessels. It is characterized by necrotizing granulomatous inflammation [1]. As per the 2012 Chapel Hill Consensus Conference on Modern Nomenclature of Systemic Vasculitis, it is categorized as antineutrophil cytoplasmic antibody (ANCA)-associated vasculitis (AAV), in conjunction with microscopic polyangiitis (MPA) and eosinophilic granulomatosis with polyangiitis (EGPA) (also known as Churg-Strauss syndrome) [2]. Although the disease might proceed to cause serious consequences, the upper and lower respiratory tracts are frequently affected by its initial signs. In over 80% of cases, renal problems such as glomerulonephritis develop. GPA can worsen rapidly, so early diagnosis and treatment are essential to prevent organ damage or failure [3]. Glucocorticoids such as prednisone are used in combination with other medications that suppress the immune system to control inflammation. Timely recognition and initiation of appropriate therapy are essential to improve outcomes in GPA. This case report presents an unusual clinical manifestation of GPA in a 54-year-old female presenting with alveolar hemorrhage without renal involvement.

## Case presentation

**Figure 1:**
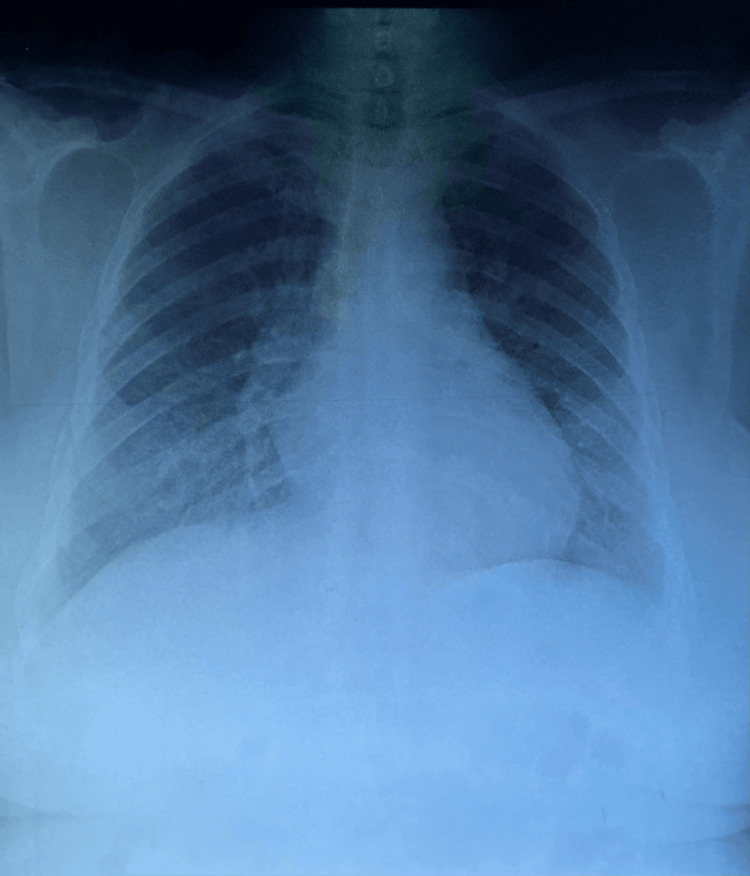
Posteroanterior chest radiograph demonstrating bilateral interstitial syndrome The image shows diffuse bilateral infiltrative opacities predominantly in the middle and lower regions.

Here, we present the case of a 54-year-old female patient, with no notable pathological history. The patient was admitted to the hospital for etiological assessment of diffuse interstitial lung disease, associated with chronic anemia in the context of altered general condition. Initially, the patient was treated with antibiotic therapy (C3G + levofloxacin) combined with short-term corticosteroid therapy without improvement and was then referred to us for an etiological assessment of her diffuse interstitial lung disease. On examination, the patient reported exposure to poultry (canaries and parakeets) and essential oil vapors for 20 years, stage II dyspnea, and low-grade hemoptysis for one year. Physical examination on admission revealed a conscious patient, with pale skin and mucous membranes, discolored conjunctiva, tachycardia at 120 beats/minute, and saturation at 89% AA. Ear, nose, and throat (ENT), pleuropulmonary, and cardiac examinations were normal. A frontal chest X-ray revealed an interstitial syndrome with bilateral infiltrative opacities (Figure 1). 

**Figure 2:**
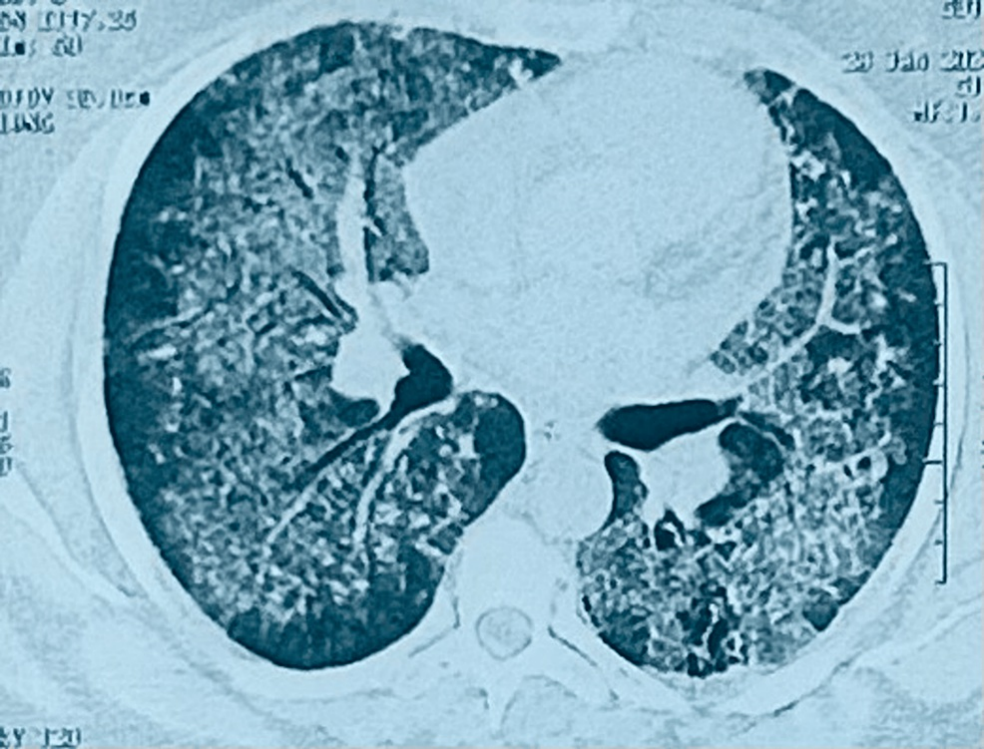
Chest computed tomography scan in the parenchymal window showing alveolar hemorrhage Chest computed tomography scan showing bilateral diffuse ground glass associated with septal and non-septal thickening, giving a crazy paving appearance, more marked in the upper lobes with sparing of the subpleural region, suggesting alveolar hemorrhage.

A biological workup showed normochromic normocytic regenerative anemia, negative C-reactive protein, normal white blood count, elevated D-dimers, negative 24-hour proteinuria, and normal blood urea (Table 1). Sputum tests for mycobacteria were negative. The anti-fowl antibody test (fluorescent enzyme immunoassay (FEIA)) was positive at 6 mg/L, which may be consistent with bird breeder's lung disease or a non-specific reactivity of the chicken antigen test. An immunological workup revealed the presence of ANCA with cytoplasmic fluorescence (c-ANCA) (>1/20 U), and p-ANCA was negative, strongly suggesting GPA. The rest of the immunological workup, particularly antinuclear antibodies (ANA), was positive with a weak homogeneous type (1/160), while anti-DNA antibodies were negative. Chest computed tomography (CT) showed diffuse bilateral ground glass associated with septal and non-septal thickening, giving a crazy paving appearance, more marked in the upper lobes with sparing of the subpleural region, suggestive of alveolar hemorrhage (Figure 2).

**Table 1:** Laboratory investigations WBC, white blood count; c-ANCA, antineutrophil cytoplasmic antibodies; p-ANCA, perinuclear anti-neutrophil cytoplasmic antibodies; ANA, antinuclear antibodies; anti-DNA, anti-double-stranded antibodies; FEIA, fluorescent enzyme immunoassay

Test	Observed value	Reference range
Hemoglobin	7.1 g/dL	12-16 g/dL
Mean corpuscular volume	79.9 fL	80-100 fL
Average corpuscular hemoglobin content	24.1 pg	27-32 pg
D-dimers	3,019.33 ng/mL	<500 ng/mL
WBC	7,390/uL	4,000-10,000/uL
C-reactive protein	7.15 mg/l	0-5 mg/L
Blood urea	6.9 mg/L	7-20 mg/dL
24-hour proteinuria	0.008 g/L	401-1,300 g/L
Sputum tests for mycobacteria	Negative	-
Angiotensin-converting enzyme	29.4 UI/L	13.30-63.90 UI/L
Anti-fowl antibody test (FEIA)	6 mg/L	<40 mg/L
c-ANCA	>1/20 U (positive)	1/20
p-ANCA	Negative	1/20
ANA	1/160	1/80
Anti-DNA	<16 UI/mL	>24 UI/mL

**Figure 3:**
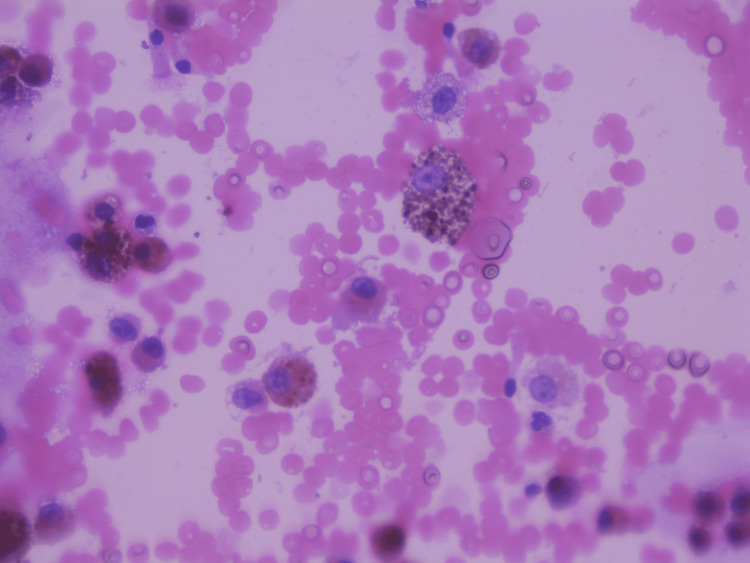
Bronchoalveolar lavage showing hemosiderosis Bronchoalveolar lavage showing predominantly macrophagic histiocytic alveolitis, with hemosiderosis. Perls' staining revealed intense hemosiderin pigmentation, leaving the histiocyte nuclei visible. This pigmentation is rated mostly at 3, according to the Golde score, and was assessed on 100 histiocytic cells.

Bronchoscopy with bronchoalveolar lavage (BAL) and Perls' staining revealed predominantly histiocytic macrophagic alveolitis with hemosiderosis. The diagnosis of GPA was based on the presence of suggestive clinical signs, notably the notion of hemoptysis, anemia, and suggestive appearance in the scan: multifocal ground glass with respect for the periphery, association of septal lines (crazy paving), and the presence of siderophages in the BAL. The diagnosis was also based on the American College of Rheumatology (ACR)/European League Against Rheumatism (EULAR) 2022 classification criteria: the presence of c-ANCA and chest X-ray abnormalities (5 points) (Figure 3).

The patient received high-dose intravenous methylprednisolone corticosteroids (1,000 mg/day) for three consecutive days. Treatment was subsequently changed to oral prednisone (1 mg/kg), with progressive dose reduction until a dose of 10 mg/d was maintained. After high-dose treatment, asthenia and cough disappeared, with improvement in his oxygen saturation. After the definitive diagnosis of granulomatosis with polyangiitis, corticosteroids were eliminated, and treatment continued. No follow-up radiological examinations were conducted at the current time. The subsequent course was favorable, with good improvement in symptoms.

## Discussion

GPA is a serious systemic disease of undetermined origin, characterized by its triple tropism of ENT, lung, and kidney. Its diagnosis is based on clinical, biological, and histological evidence. GPA remains a rare disease, generally occurring in patients between 40 and 50 years of age [4], and is rarely seen in the elderly, as evidenced by the paucity of published geriatric series [5]. Diagnosis is often made later in this age group, with various series in the literature showing an average delay of 5-13 months after the onset of the first symptoms [6]. This long delay was observed in our first patient, who had been suffering from lingering pulmonary symptoms for a year. This generally long delay in diagnosis in the elderly could be explained by the atypical nature of the initial presentation, which may make the diagnosis more difficult, and the existence of a comorbidity associated with this age group. The inaugural signs of GPA in adults are multiple and may include ENT manifestations in 73%-83% of cases, articular manifestations in 20%-60% of cases, and pulmonary manifestations in 20%-45% of cases. Screening for renal impairment is of major interest in GPA, given its prognostic impact on patient survival. The prevalence of nephropathy in GPA is variable, ranging from 45% to 90% of cases depending on the series and depending on the origin of patient recruitment, the definition used to label renal disease, and the length of follow-up. Indeed, in Hoffman's series, only 18% of patients had nephrological signs on initial diagnosis of GPA, but after a few years' follow-up, nearly 80% developed renal involvement. Various studies have reported an increased prevalence of renal involvement in GPA in elderly subjects; renal involvement was absent in our patient. In a geriatric series published by Fauchais et al. [7], including 11 patients aged over 60 and followed for GPA, ENT involvement was observed in 91% of cases, and pulmonary involvement was also frequent, affecting 82% of cases [8]. Advanced age is also a significant prognostic factor in GPA and is an important factor to take into account, particularly when choosing immunosuppressive therapy, since several studies seem to suggest that the high mortality of patients over 65 years of age is largely secondary to the side effects of immunosuppressive drugs [9]; in our patient's case, the course after corticosteroid treatment was favorable.

## Conclusions

Granulomatosis with polyangiitis is a severe systemic disease. Its diagnosis, not always easy for the clinician unaware of this type of disease, is based on non-specific but often quite noisy clinical signs (particularly ENT and pulmonary), characteristic histological abnormalities, and the almost constant presence of a specific autoantibody, anti-PR3 c-ANCA. This paper documents an atypical initial presentation of GPA in a 54-year-old female, supported by ANCA positivity, positive findings on scans, and the presence of siderophages in bronchoalveolar lavage (BAL). Prompt identification of GPA demands a heightened level of suspicion, especially when facing atypical presentations. Through meticulous differential diagnosis involving infectious diseases, neoplasia, and immunological disorders, we concluded that the accurate diagnosis is granulomatosis with polyangiitis. Accelerating the diagnostic process is essential to facilitate timely and efficient treatment and mitigate the progression of the disease.

## References

[1] Garlapati P, Qurie A (2022). Granulomatosis with polyangiitis. https://www.ncbi.nlm.nih.gov/books/NBK557827/.

[2] Jennette JC (2013). Overview of the 2012 revised International Chapel Hill Consensus Conference nomenclature of vasculitides. Clin Exp Nephrol.

[3] Idolor ON, Guraya A, Muojieje CC (2021). Renal involvement in granulomatosis with polyangiitis increases economic health care burden: insights from the National Inpatient Sample database. Cureus.

[4] Scapa JV, Fishbein GA, Wallace WD, Fishbein MC (2018). Diffuse alveolar hemorrhage and pulmonary vasculitides: histopathologic findings. Semin Respir Crit Care Med.

[5] Berriche O, Hammami S, Ammari FL, Alaya W, Kessomtini W, Chebbi W (2015). [Granulomatosis with polyangiitis in the elderly: report of two cases and review of literature]. Pan Afr Med J.

[6] Lichtenberger JP 3rd, Digumarthy SR, Abbott GF, Shepard JA, Sharma A (2014). Diffuse pulmonary hemorrhage: clues to the diagnosis. Curr Probl Diagn Radiol.

[7] Weiner M, Goh SM, Mohammad AJ (2015). Outcome and treatment of elderly patients with ANCA-associated vasculitis. Clin J Am Soc Nephrol.

[8] Nasser M, Cottin V (2018). Alveolar hemorrhage in vasculitis (primary and secondary). Semin Respir Crit Care Med.

[9] Lara AR, Schwarz MI (2010). Diffuse alveolar hemorrhage. Chest.

